# New silicate-substituted hydroxyapatite materials doped with silver ions as potential antifungal agents

**DOI:** 10.1186/s12866-023-02930-w

**Published:** 2023-07-18

**Authors:** Agata Piecuch, Sara Targońska, Justyna Rewak-Sorczyńska, Rafał Ogórek, Rafal J. Wiglusz

**Affiliations:** 1 Department of Mycology and Genetics, University of Wrocław, Przybyszewskiego 63/77, Wroclaw, 51-148 Poland; 2 Institute of Low Temperature and Structure Research, Polish Academy of Sciences, Okolna 2, Wroclaw, 50-422 Poland

**Keywords:** Hydroxyapatites, Silver ions, Yeasts, Antifungal agent, Opportunistic pathogens

## Abstract

**Background:**

Hydroxyapatites (HAp) are widely used as medical preparations for e.g., bone replacement or teeth implants. Incorporation of various substrates into HAp structures could enhance its biological properties, like biocompatibility or antimicrobial effects. Silver ions possess high antibacterial and antifungal activity and its application as HAp dopant might increase its clinical value.

**Results:**

New silicate-substituted hydroxyapatites (HAp) doped with silver ions were synthesized via hydrothermal methods. The crystal structure of HAp was investigated by using the X-ray powder diffraction. Antifungal activity of silver ion-doped HAp (with 0.7 mol%, 1 mol% and 2 mol% of dopants) was tested against the yeast-like reference and clinical strains of *Candida albicans*, *C. glabrata*, *C. tropicalis*, *Rhodotorula rubra*, *R. mucilaginosa*, *Cryptococcus neoformans* and *C. gattii*. Spectrophotometric method was used to evaluate antifungal effect of HAp in SD medium. It was shown that already the lowest dopant (0.7 mol% of Ag^+^ ions) significantly reduced fungal growth at the concentration of 100 µg/mL. Increase in the dopant content and the concentration of HAp did not cause further growth inhibition. Moreover, there were some differences at the tolerance level to Ag^+^ ion-doped HAp among tested strains, suggesting strain-specific activity.

**Conclusions:**

Preformed studies confirm antimicrobial potential of hydroxyapatite doped with silver. New Ag^+^ ion-HAp material could be, after further studies, considered as medical agent with antifungal properties which lower the risk of a surgical-related infections.

## Background

Despite the fact that fungal infections are incomparably less abundant in the population than bacterial ones, they could pose a risk towards people, especially immunocompromised patients [1]. Fungi can affect different sites of human body, hence various types of fungal infections can be distinguished: systemic, superficial, cutaneous and subcutaneous [2]. Among fungi that are mostly responsible for infections in humans are genera like: *Aspergillus*, *Candida*, *Cryptococcus*, *Rhizopus*, *Mucor* and *Rhizomucor* [3].There are also numerous emerging fungal pathogens that are reported to cause more and more infections, such as: non-*Aspergillus* molds (e.g., *Fusarium*) as well as yeast and yeast-like fungi (e.g., *Rhodotorula*, *Trichosporon* and *Malassezia*) [4]. Over 80% of candidiasis are caused by *C. albicans*, and the most prevalent non-*Candida albicans* candidiasis (NCAC) are attributed to *C. glabrata*, *C. parapsilosis*, *C. tropicalis*, *C. krusei* and *C. dubliniensis* [1]. Cryptococcosis, which could affect lungs, skin and central nervous system, is mainly caused by *Cryptococcus gatti* and *Cryptococcus neoformans* [1]. Moreover, *Candida* and *Cryptococcus* are frequently found in multispecies biofilms, alongside with various bacteria, and they cause mixed infections [1]. Among *Rhodotorula* genus, the most prevalent species are *R. glutinis*, *R. mucilaginosa*, *R. rubra* and *R. minuta*. The most common infections caused by *Rhodotorula* are detected in the blood stream and in the central nervous system [3]. Apart from primary fungal infections there are also reports describing occurrence of co-infections and secondary infections in the patients suffering from severe respiratory viral infections, like COVID-19. Among such pathogens are species belonging to the genera of *Aspergillus* and *Candida* [4]. Such infections in immunocompromised patients may lead to the deterioration of health and even increase the mortality rate [4]. This is why it is so important to cure fungal infections properly.

Apart from widely known and broadly used antifungal drugs, like fluconazole, there are also alternative therapies, that still gain a lot of interest. Among such therapies is the usage of silver ions (Ag^+^) and silver-based nanoparticles [5]. Despite the fact that testing the activity of silver ions in vitro is associated with many difficulties, like complexation of Ag^+^ ion with medium components [6], there are a lot of research describing its activity against bacteria, fungi and viruses [5, 7]. Among the observed effects of silver on fungal cell are membrane lipid bilayer damage, which results in the leakage of ions (like K^+^) and nutrients (glucose) from the cell, and pore formation in the membrane, which can lead even to its death. The other mechanism is based on the reactive oxygen species (ROS) generation. There are also other reported changes: surface morphology alteration, changes in membrane fluidity, ergosterol content and fatty acid composition, disintegration of cell wall, surface protein damage, nucleic acid damage and blockage of proton pumps. Moreover, silver nanoparticles inhibit yeast budding [5, 8–10]. It is hypothesized that the effect on fungal cell is dependent on both Ag^+^ ions and AgNPs simultaneously and the exact mechanism has to be furtherly studied [8]. Nevertheless, fungal cells developed few mechanisms that prevent them from harmful action of Ag^+^ and other silver-based compounds: modification, sequestration, facilitated efflux and reduced influx. For example, *Candida* species reduce Ag^+^ ions to nonharmful nanoparticles within the cell [5]. Moreover, one of the most effective ways to reduce the impact of chemicals on microbial cell is biofilm formation. Among strains that were tested in the present research the biggest biofilm producer is *C. glabrata* (especially the strains 137 and 327) [11].

Silver nanoparticles are the most commercially used nanomaterials [8]. Methods of chemical synthesis allow to obtained materials of desired features, like shape, size and morphology of the grains. Such features highly influence the effectiveness of AgNPs and generally the smaller nanoparticle is the better it penetrates through the cell’s barriers [8]. Silver ions are also used as dopants in more complex nanomaterials, like hydroxyapatite Ca_10_(PO_4_)_6_(OH)_2_ (HAp). HAp is known as a nontoxic, biocompatible, and biodegradable substance that naturally occurs in the human body, as a component of teeth and bones. Due to its osteo-regenerative properties it is also used as porous scaffold in bone grafts or tissue filler [7]. Calcium cation in its formula could be partly substituted with other ions, including silver, which allows to create nanomaterials with antimicrobial potential. The recommended silver dopant in hydroxyapatite varies between 0.5 and 2 mol% [7]. Moreover, the addition of silver to the hydroxyapatite structure improve its physicochemical properties, like crystallinity and solubility [12]. In the structure of HAp not only Ca^2+^ ions could be substituted but anionic modifications could be implemented as well. Phosphate groups (PO_4_^3-^) could be partially substituted with silicate groups (SiO_4_^3-^) which also modifies the properties of the material, for example causing the increase in the surface area/volume ratio [12]. Moreover, the Ag^+^/Si-HAp is reported to cause reduced bacterial adhesion which proves that such compounds have great potential [13].

Bone regeneration materials commonly consist of hydroxyapatite-based products (HAp). Aside from its advantage - osteogenic properties, HAp also provides a survivable environment for pathogens. An effective strategy to prevent the initial adhesion of microorganisms and the colonization of biofilms could be for example the introduction of bioactive antimicrobial thin films on the bone implants’ surface [14, 15]. The main goal of present research is to evaluate the antifungal activity of newly synthesized silver-doped silicate-modified hydroxyapatites against yeast-like species of clinical relevance. These studies could provide preliminary results for the future potential application of Ag^+^-doped Hap in combating surgery-associated infections. It is predicted that the presented materials could be promising for the construction of bone regeneration materials, which combine bioactive characteristics with antimicrobial properties.

## Results and discussion

Hydroxyapatites (HAp) due to their high biocompatibility are widely used as biomaterials e.g., as bone or teeth replacements. The incorporation of these materials during surgical procedures carries the risk of an infection. Although such nosocomial infections are mainly associated with bacteria, the incidence of fungal contamination is also frequent [16]. Lowering the risk of microbial invasion in patients is of great importance and incorporation of antimicrobial substances in the hydroxyapatites is one way to achieve it. Numerous metals were shown to possess antimicrobial activity, examples being zinc or copper, however silver is the best known for its antimicrobial properties [17–20].

### Characterization

**Fig. 1:**
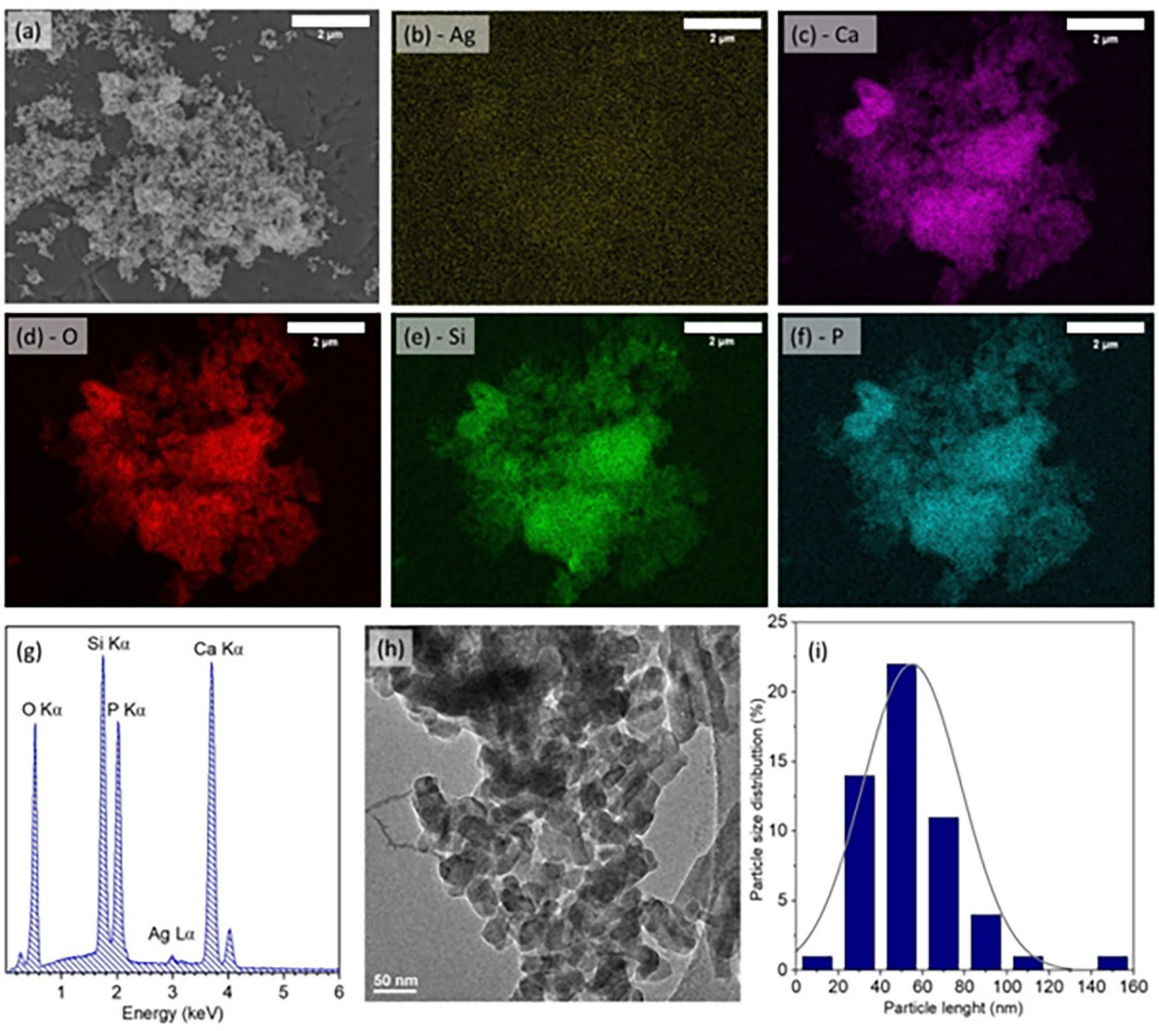
The SEM image (**a**), EDS element mapping (**b-f**), EDS element analysis (**g**), TEM image (**h**), and particle size distribution (**i**) of 2Ag-HAp.

The morphology, shape and element mapping of tested samples were collected by SEM imaging and the results are shown in Fig. 1a-f. According to SEM images, the powders obtained are irregularly rounded in shape and tend to agglomerate. Element mapping confirms the random distributions of dopant ions, silicate, and phosphate groups in the obtained powder. The concentration of Ag, and Si were calculated by the EDS analysis (Fig. 1g). It was confirmed that Ag concentration equaled to 0.07; 0.11, and 0.20 mol. The silicate group number in each of the samples was approximately 3. Consequently, the chemical formula of tested samples is: Ca_10-x_Ag_x_(PO_4_)_3_(SiO_4_)_3_(OH)_2_, where x = 0.07; 0.11, and 0.20 mol. In the presented paper samples are named: 0.7Ag-HAp; 1Ag-HAp, and 2Ag-HAp, respectively. The number in the abbreviation is associated with the Ag^+^ ion molar concentration. The content of dopant ions was calculated using the following equation:

1 $${{\rm{X}}_{{\rm{n/x}}}}{\rm{[mol\% ] = }}\frac{{{\rm{mol\% X}} \cdot {\rm{10}}}}{{{\rm{mol\% }}\,{\rm{(C}}{{\rm{a}}^{{\rm{2 + }}}}{\rm{ + }}\,{\rm{A}}{{\rm{g}}^{\rm{ + }}}{\rm{)}}}}$$

The amount of silicate group was calculated by the equation:

2 $${\rm{(Si}}{{\rm{O}}_{\rm{4}}}{\rm{)}}_{\rm{y}}^{{\rm{4}} - }{\rm{[mol\% ]}}\,{\rm{ = }}\,\frac{{{\rm{mol\% S}}{{\rm{i}}^{{\rm{4 + }}}} \cdot {\rm{6}}}}{{{\rm{mol\% }}\,{\rm{(S}}{{\rm{i}}^{{\rm{4 + }}}}\,{\rm{ + }}\,{{\rm{P}}^{{\rm{5 + }}}}{\rm{)}}}}$$

The TEM images were used to designate the particles shape and size (see Fig. 1h). According to the TEM images, the particle length was measured, and Fig. 1i displays the histogram of the particle size distribution. The particle size is in a range of 20–80 nm.

**Fig. 2:**
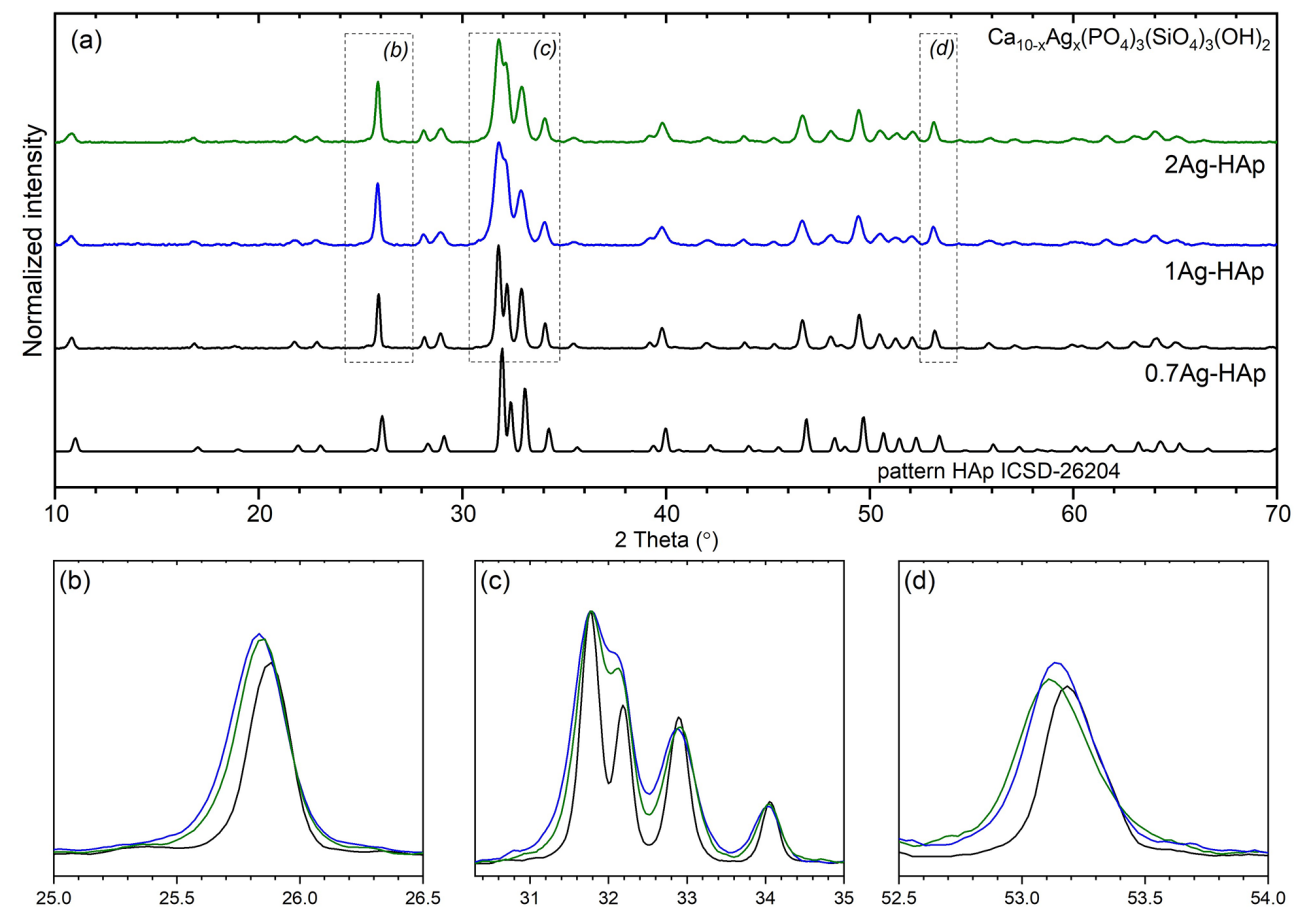
(**a**) X-ray diffraction pattern of silicate-substitution hydroxyapatite doped with Ag^+^ ions; (**b**) diffraction pattern of reflection (002); (**c**) reflections (211), (112), (300), and (202); (**d**) reflection (004)

Analysis of the crystal structure was conducted based on X-ray diffraction (Fig. 2a). By comparing the products to the reference standard hexagonal hydroxyapatite ICSD-26,204 [21], the single phase composition of the final products was confirmed. The diffraction peaks that correspond to hydroxyapatite structure are located at 25.9° (002), 31.8° (211), 32.1° (112), 32.9° (300) and 34.1˚ (202); 40.0° (310); 46.7° (222); 49.5° (210); 53.2° (004) (Fig. 2b and c). The crystallographic planes are listed in the brackets. The obtained materials were found to have the dopant ions completely incorporated into the structure of the silicate-substituted apatites. With the increasing dopant concentration, the diffraction peaks are broadened what is related to the particle-size-effect (see Fig. 2c), resulting in physicochemical properties of the obtained materials [22, 23].

According to the Debye-Scherrer formula, average crystallite sizes (D) were calculated using x-ray diffraction patterns (Eq. 3).

3 $$D= \frac{K{\uplambda }}{{\upbeta }\text{c}\text{o}\text{s}{\Theta }}$$

Where: D - average crystallite sizes; K - Scherrer constant (0.89), λ - x-ray wavelength (0.15406 nm), β - FWHM (°) of the three selected reflections. For the purpose of the approximation, the following peaks were used: 34.1˚ (202); 46.7° (222); 49.5° (210). In the case of a lower concentration of Ag^+^-dopant, the average crystallite size is the largest, 55.6 ± 9.9 nm. Samples with 1 mol% and 2 mol% of Ag^+^ ions are characterized with similar average size, 30.9 ± 4.2 nm and 35.6 ± 5.5 nm, respectively. The differences of average gain size are visible on the x-ray pattern. The peaks’ maximum are shifted to the lower 2theta angles when the gain size is smaller. It is observed in case of investigated pattern (see Fig. 2b and d).

**Fig. 3:**
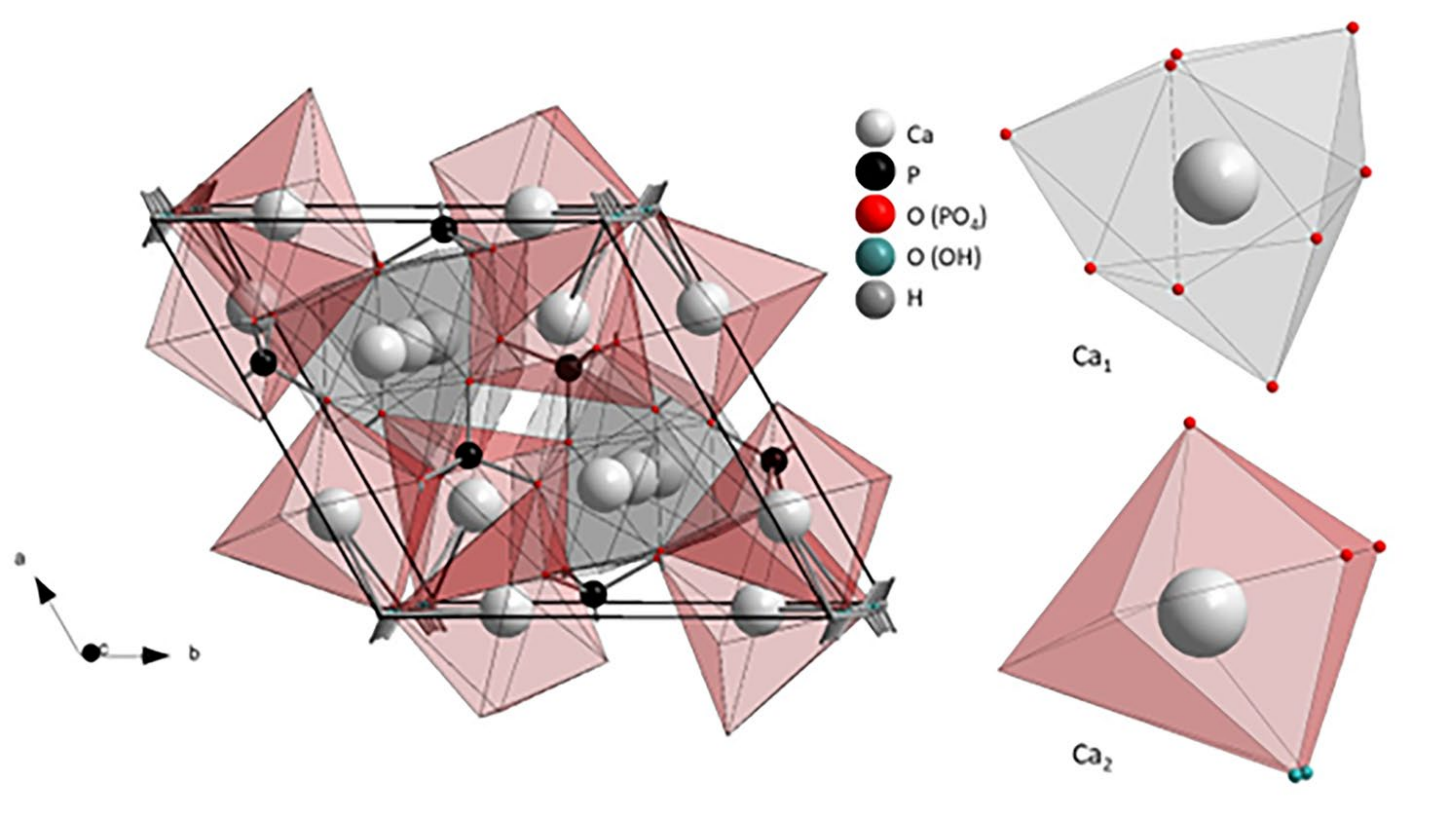
Representation of pure HAp cell unit, Ca(1), and Ca(2) polyhedrons

The apatite molecules are crystalized with hexagonal symmetry (*P6*_*3*_*/m*) where Ca^2+^ ions anadoccupy two structurally distinct crystallographic sites. The Ca(1) side is surrounded by 9 oxygen atoms from phosphate groups creating the *4f* site. The Ca(2) atoms are placed in *6 h* site, in the close environment of 6 oxygen atoms from the phosphate group, and one from the hydroxyl group. Figure 3 shows the unit cell representation and coordination polyhedrons of calcium sites.

**Fig. 4:**
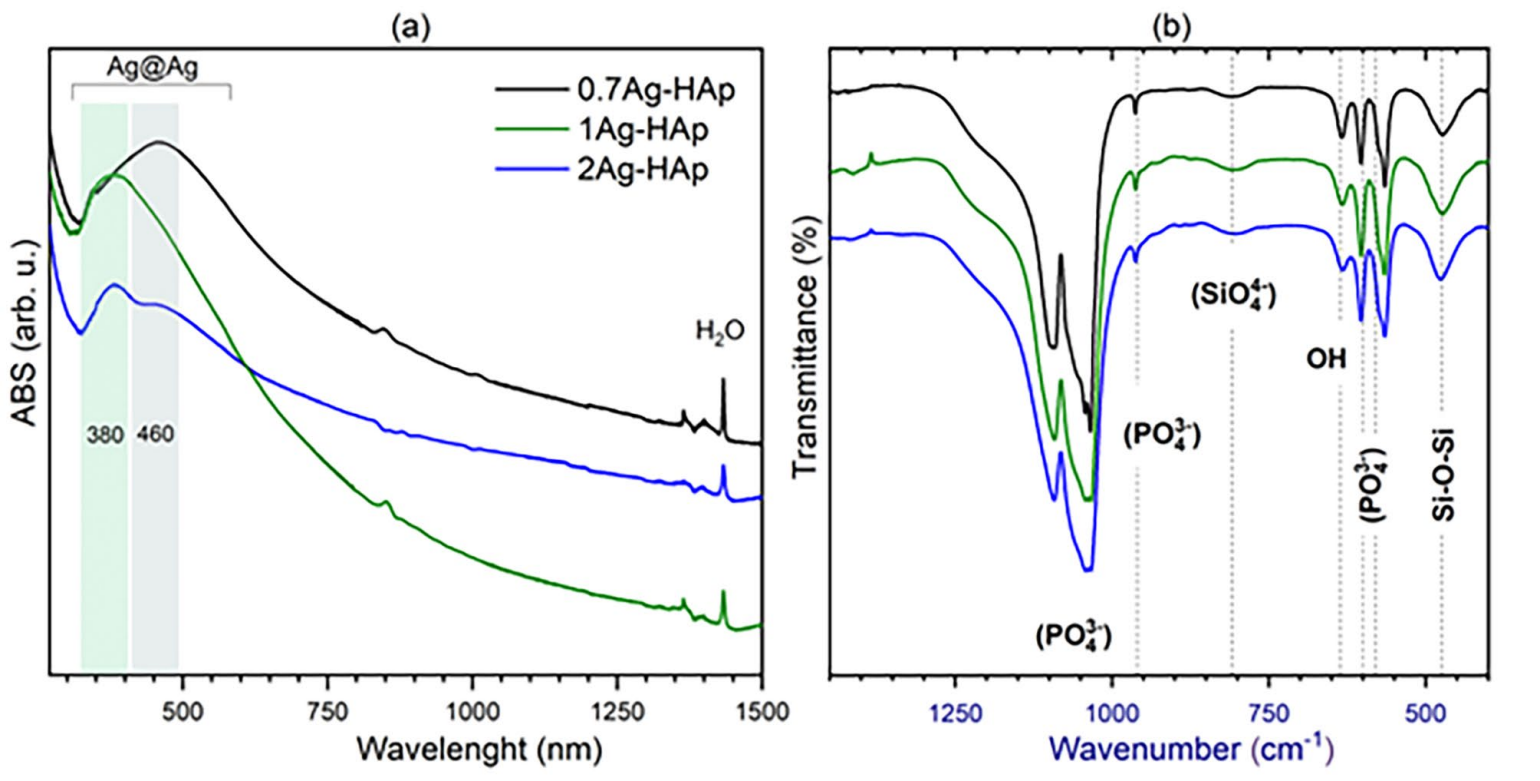
UV absorption (**a**), FT-IR spectra (**b**)

The UV absorption, as well as FT-IR spectra were collected (see Fig. 4). Two broad peaks are detected on the UV spectra, centered at 380 and at 460 nm. Those peaks are related to the nanosized silver plasmons (Ag-NPs), presented as a consequence of the Ag^+^ ions reduction to silver (Ag^0^) [24].

The FT-IR spectra were collected in the range of wavenumbers of 1500–400 cm^-1^ to perceive the characteristic peaks associated with the hydroxyapatite molecule vibrational modes. The triply degenerated antisymmetric stretching vibration of phosphonate group *ν*_*3*_*(PO*_*4*_^*3-*^*)* was detected as two strong peaks at 1095 cm^− 1^ and at 1045 cm^− 1^. The band at 963 cm^− 1^ belongs to the non-degenerated symmetric stretching modes of *ν*_*1*_*(PO*_*4*_^*3-*^*)*. The triply degenerated vibrations of *ν*_*4*_*(PO*_*4*_^*3-*^*)* were visible as two peaks at 608 cm^− 1^ and at 588 cm^− 1^. The broad peak at the 811 cm^-1^ was marked to the ν_3_*(SiO*_*4*_^*4-*^*)* vibration. Stretching vibration of Si–O–Si was located at 477 cm^-1^. The Si-O symmetric stretching vibration is not visible as the single peak, because of overlapping by the similarly located P-O symmetric stretching mode. The presence of the OH^-^ group in the hydroxyapatite crystal structure is confirmed by the band located at 634 cm^-1^ [25].

### Antifungal activity

Hydroxyapatites are commonly used in various clinical fields, thus enhancing their physicochemical and biological properties by structure modification is widely studied. Doping of hydroxyapatites with silver ions is well documented and it has been proved that those substances are active against various gram-positive and gram-negative bacteria [26]. Much less is known about their antifungal properties. Ag-doped HAp nanoparticles were active against *Candida kruzei*, both planktonic cells and biofilms [27, 28]. Similarly, Elbasuney et al. showed high efficiency of Ag^+^ ion-doped HAp on *C. albicans* and *C. neoformans*, as well as its antibiofilm activity [29]. On the other hand, the antimicrobial activity of silica-modified hydroxyapatites remains poorly understood, thus our research focused on those materials. Our previous studies on Si-HAp concerned their antibacterial effect against gram-negative bacteria and it was shown that zinc doping caused an increase in antibacterial activity, while Zn^2+^-HAp unmodified with Si had no or little effect [18, 30]. No evidence of antifungal activity of Si-HAp was previously published. To explore antimicrobial activity of Si-HAp, in present work the newly synthesized Si-hydroxyapatites were doped with different amount of silver ions and tested for their antifungal activity against various yeast-like strains, both clinical isolates and the reference ones. Those fungi are opportunistic pathogens that pose a threat of an infection in people with an impaired immune system, thus, designing new approaches to combat such pathogens is an important issue. Since HAps are materials used e.g., for bone regeneration, its application links to the surgical procedure. Improvement of their antimicrobial properties by structure modification might lower the risk of a surgical associated infection by fungi. *C. albicans* is the best known cause of the candidiasis, however an increasing occurrence of non-albicans infections is observed, *C. glabrata* and *C. tropicalis* being the most common [31, 32]. Other yeast-like opportunistic pathogens are also of great clinical significance. *C. neoformans* is a frequent cause of cryptococcal meningoencephalitis [33]. Species of *Rhodotorula* genus are also the emerging pathogens e.g., *R. mucilaginosa* being frequently associated with central venous catheter infections and *R. rubra* being a cause of meningitis [34, 35].

**Fig. 5:**
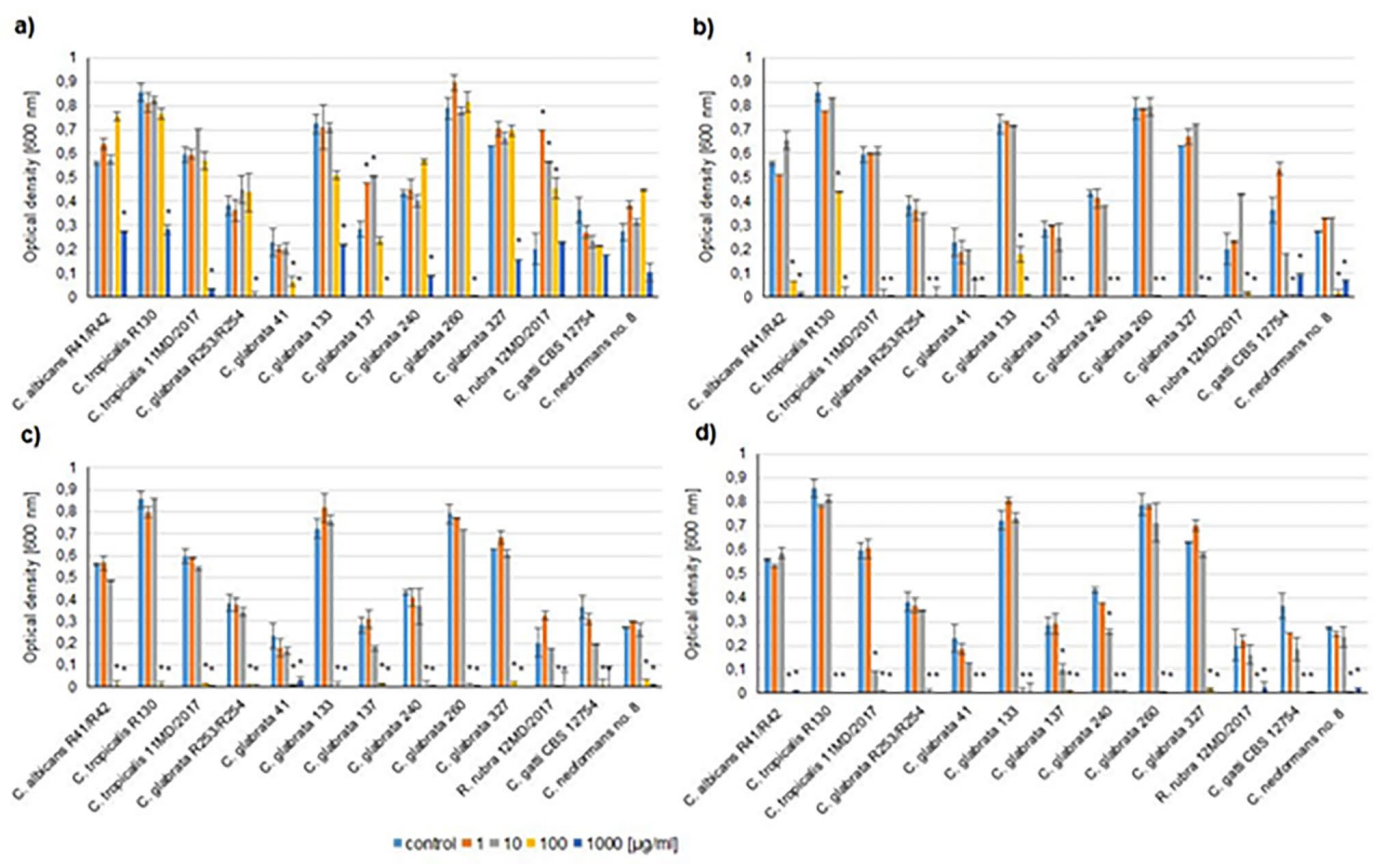
The inhibition of fungal growth of Ag-doped silica-modified hydroxyapatites (HAp) against clinical strains. **a**) – un-doped silica-HAp; **b**) – 0.7 mol% Ag^+^; **c**) 1 mol% Ag^+^; **d**) 2 mol% Ag^+^. Mean ± SD. * statistical significance (p < 0.05; ANOVA)

It was shown that among clinical isolates already 0.7 mol% of Ag^+^ ion doping caused almost complete growth reduction at the concentration of 100 µg/mL (p < 0.005), while un-doped HAp showed no significant effect (Fig. 5a and b). Two non-albicans *Candida* strains (*C. tropicalis* R130 and *C. glabrata* 133) exhibited higher tolerance to 0.7 mol% Ag^+^-HAp, however their growth was still significantly reduced when compared to the control (untreated cells) and un-doped HAp (Fig. 5a and b).

The increase in dopant content to 1 mol% did not cause any further growth inhibition, except for two above-mentioned non-albicans strains, which growth was inhibited at the level similar to the remaining fungi (Fig. 5c).

Further elevation of dopant amounts up to 2 mol% resulted in the growth reduction at the lower concentration (10 µg/mL) but this effect was observed only in the case of three clinical isolates: *C. tropicalis* 11MD/2017 (growth reduction by about 80%), *C. glabrata* 137 and *C. glabrata* 240 (inhibition by about 50% and 30%, respectively). The increase of the concentration to 100 µg/mL caused almost complete growth reduction of all tested strains, as it was observed in the case of the lower dopant contents (Fig. 5d).

**Fig. 6:**
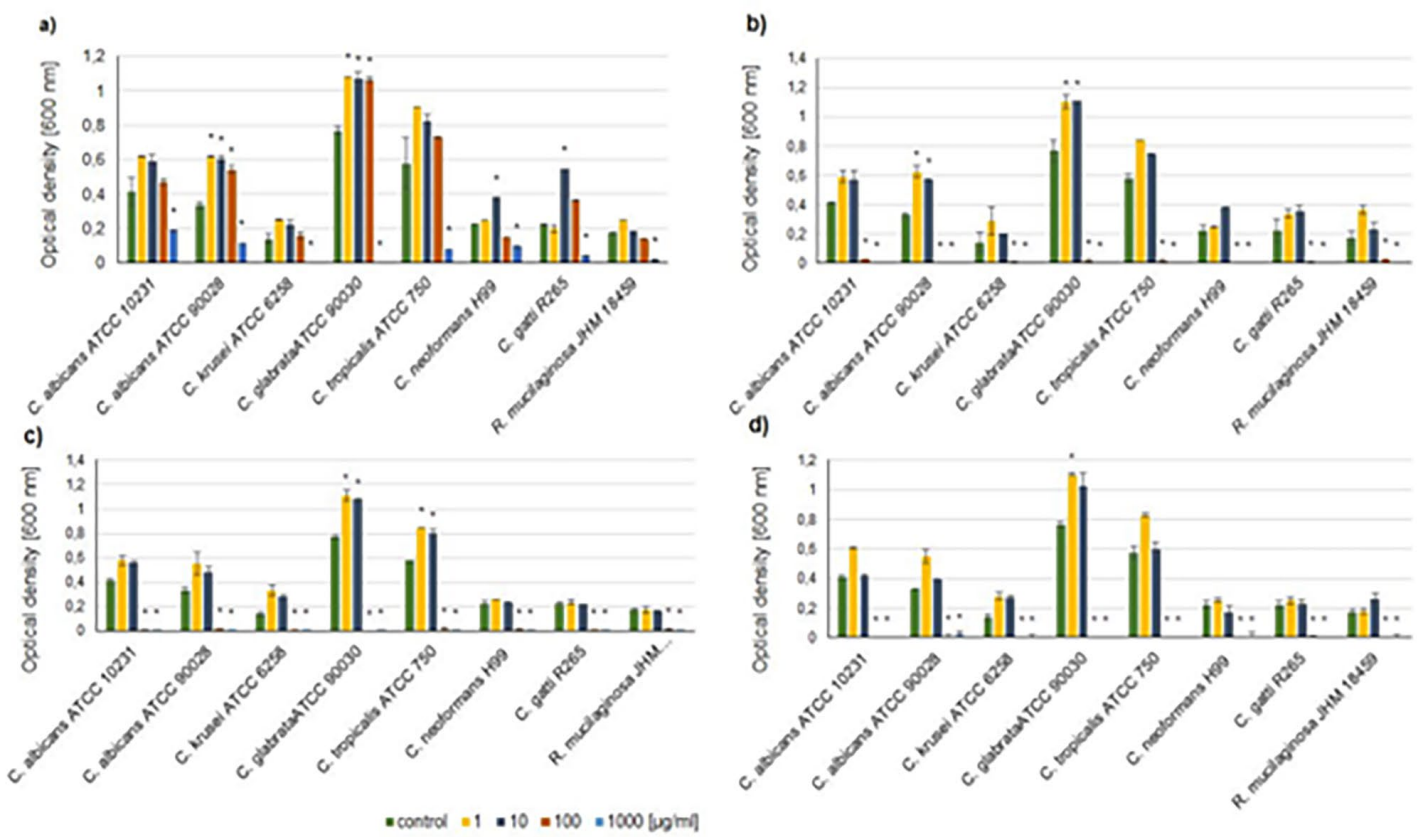
The inhibition of fungal growth of Ag-doped silica-modified hydroxyapatites (HAp) against reference strains. **a**) – un-doped silica-HAp; **b**) – 0.7 mol% Ag^+^; **c**) 1 mol% Ag^+^; **d**) 2 mol% Ag^+^ ions. Mean ± SD. * statistical significance (p < 0.05; ANOVA)

Similar tendencies were noted for the reference yeast-like strains – the higher concentrations (100 and 1000 µg/mL) of all Ag^+^ dopants (0.7 mol%, 1 mol% and 2 mol%) caused high growth reduction. The growth of certain strains, however, was stimulated by the lower concentration of Ag-doped HAp, especially in the case of *C. glabrata* ATCC90030 (Fig. 6).

Interestingly, some fluctuations in fungal growth were observed also when un-doped HAp was applied. The highest concentration (1000 µg/mL) caused growth inhibition of the most fungal strains. On the other hand, some of the tested fungi were stimulated to grow when the lower concentration of HAp was applied. This effect was observed especially for *C. glabrata* 137 and *R. rubra* 12MD/2017, as well as the reference strain of *C. glabrata* (Figs. 5a and 6a).

The studies of antifungal activity of silver doped HAp showed that the lowest amount of Ag^+^ ions exhibited activity against tested strains, however the level of fungal tolerance to tested HAps varied among fungi. The silver ions and silver nanoparticles possess multiple effects on fungal cells, related mainly to the damage to plasma membrane and cell wall, as well as an oxidative stress. It was also shown that due to AgNP action ABC transporters were inhibited and transcription of genes related to energy utilization was reduced in mycotoxin-producing fungi [36]. Resistance mechanisms in fungi include reduction of ions to nanoparticles, sequestration by metallothionein and glutathione, efflux and reduced influx [5]. Among tested strains, three of the clinical isolates: *C. albicans*, *C. tropicalis* and one of *C. glabrata* strains showed higher level of tolerance to Ag^+^-doped HAp. On the other hand, corresponding reference species were sensitive to hydroxyapatites, suggesting that the clinical strains possibly gained some resistance mechanisms to silver. Interestingly, among non-*Candida* strains slightly higher tolerance was observed in clinical isolates of cryptococci (Figs. 5 and 6).

## Conclusions

Hydroxyapatatites are excellent materials for medical application and improving its clinical value is of great interest. Presented in this work novel silver-doped silicate-substituted hydroxyapatites were characterized and tested regarding their antifungal activity against a set of yeast-like fungi, both reference and clinical strains. The antifungal effect of Ag^+^-HAp against those opportunistic pathogens of humans was exhibited already at the lowest dopant amount. These promising preliminary results point at the potential of silver-doped hydroxyapatites in the clinical application as biocompatible materials that lower the risk of the fungal nosocomial infections.

## Materials and methods

### Synthesis of silver-doped hydroxyapatite

Silver-doped silicate-substituted hydroxyapatite was synthesized via hydrothermal methods. As substrates Ca(NO_3_)_2_ 4H_2_O (99.0-103.0% Alfa Aesar), (NH_4_)_2_HPO_4_ (> 99.0% Acros Organics, AgNO_3_ (99.8%, POCH) and tetraethyl orthosilicate TEOS (> 99% Alfa Aesar) were used. Three concentrations of biologically active silver ions in the ratio of calcium ions, 0.7, 1.2, and 2.0 mol% were tested. Deionized water was used to dissolve the stoichiometric number of substrates. Substrates were mixed in the Teflon vessels. To obtain pH equal to 10, ammonia solution (25% Avantor, Poland) was used. A microwave reactor (ERTEC MV 02–02) was used to heat the hydrothermal process for 90 min at an elevated temperature (250 °C) and pressure (42–45 bar). Products were cleaned with deionized water and dried for 24 h. After that, powders were heat-treated at 600 °C, for 3 h, to obtain well-crystalized products. The general chemical formula of obtained material is: Ca_10-x_Ag_x_(PO_4_)_3_(SiO_4_)_3_(OH)_2_, where x = 0.07; 0.1, and 0.2. The hydrothermal method and process parameters were selected according to the previous investigations [37, 38].

### Materials characterization

The crystal structure was investigated by using the x-ray powder diffraction. Diffraction patterns were measured by PANalyticalX’Pert Pro X-ray diffractometer equipped with Ni-filtered Cu *Kα*_*1*_ radiation (*Kα*_*1*_ = 1.54060 Å, *U* = 40 kV, *I* = 30 mA) in the *2*θ range of 10–70˚. Match! software 3.7.0.124 version was used to analyze the measurements.

The average size, surface morphology and element content were carried out by a FEI Nova NanoSEM 230 scanning electron microscopy equipped with EDS spectrometer (EDAX GenesisXM4). Equipment was working at an acceleration voltage in the range of 3.0–15.0 kV and spots 2.5–3.0 were observed.

Fourier-transformed infrared spectra were collected by aThermo Scientific Nicolet iS50 FT-IR spectrometer equipped with an Automated Beamsplitter exchange system (iS50 ABX containing DLaTGS KBr detector), built-in all-reflective diamond ATR module (iS50 ATR), Thermo Scientific Polaris™. The HeNe laser was used to generate the infrared radiation. The investigated samples were mixed with KBr (FT-IR grade, ≥ 99% Sigma-Aldrich, St. Louis, MO, USA), and pellets were formed. FT-IR spectra were recorded at the temperature of 295 K in middle infrared range, with a spectral resolution of 2 cm^− 1^.

An Agilent Cary 5000 spectrophotometer was used to measure the absorbance spectrum with a spectral bandwidth (SBW) of 0.1 nm in the visible and ultraviolet range (240–1500 nm) at room temperature.

### Strains

For antifungal testing following reference strains and clinical isolates were used: *Candida albicans* ATCC 90,028, *C. albicans* R41/R42, *C. tropicalis* ATCC 750, *C. tropicalis* R130, *C. tropicalis* 11MD/2017, *C. glabrata* ATCC 90,030, *C. glabrata* R253/R254, *C. glabrata* 41, *C. glabrata* 133, *C. glabrata* 137, *C. glabrata* 240, *C. glabrata* 260, *C. glabrata* 327, *Rhodotorula rubra* 12MD/2017, *R. mucilaginosa* JHM 18,459, *Cryptococcus gatti* R265, *C. gatti* CBS 12,754, *C. neoformans* H99 and *C. neoformans* no. 8. All reference strains as well as *C. tropicalis* 11MD/2017 and *R. rubra* 12MD/2017 are a part of the collection in the Department of Mycology and Genetics, University of Wroclaw. Remaining clinical isolates are a kind gift from the Department of Microbiology, Medical University, Wroclaw.

### Antifungal activity

Antifungal activity of tested HAps was studied according to Rewak-Soroczyńska et al. [11]. Colloidal solutions of Ag^+^-doped hydroxyapatites (0.7 mol%, 1 mol% and 2 mol% of dopants) were prepared by sonification (Virtis Virsonic 50) in minimal SD medium (Synthetic Defined; 6.7 g/L Yeast Nitrogen Base w/o aminoacids (Difco), 20 g/L glucose) at the concentration of 10 mg/mL. The final concentration of 10, 100 and 1000 µg/mL were prepared by the serial dilution in SD medium.

Fungal strains were incubated in YPD medium (Lab Empire) at 28 ± 0.5 °C for 24 h (for *Candida* strains) or 48 h (for *Rhodotorula* and *Cryptococcus* strains). Cells were centrifuged and suspended in SD medium to obtain optical density of 0.1 and diluted 50x in SD.

The wells of the 24-well polystyrene plate were filled with SD medium (0.8 mL), solution of doped and un-doped hydroxyapatite (0.1 mL) and fungal suspension (0.1 mL). Plates were incubated at 28 ± 0.5 °C without shaking for 24 h (for *Candida* strains) or 48 h (for *Rhodotorula* and *Cryptococcus* strains). The well content was mixed, and optical density was measured using microplate reader (VarioSkan LUX, Thermo Fisher Scientific) at the wavelength of 600 nm. As a control cells untreated with hydroxyapatites were applied. As a blank clear SD medium without fungi was used. The experiment was repeated 3 times, independently. Statistical analysis of obtained results was performed using ANOVA test.

## Data Availability

All data generated or analysed during this study are included in this published article.

## References

[1] Gnat S, Łagowski D, Nowakiewicz A, Dyląg M (2021). A global view on fungal infections in humans and animals: opportunistic infections and microsporidioses. J Appl Microbiol.

[2] Kothavade RJ, Kura MM, Valand AG, Panthaki MH (2010). Candida tropicalis: its prevalence, pathogenicity and increasing resistance to fluconazole. J Med Microbiol.

[3] Spallone A, Schwartz IS (2021). Emerging fungal infections. Infect Dis Clin North Am.

[4] Roudbary M, Kumar S, Kumar A, Černáková L, Nikoomanesh F, Rodrigues CF. Overview on the prevalence of fungal infections, immune response, and microbiome role in COVID-19 patients. J Fungi. 2021;7. 10.3390/jof7090720 PMC8466761 34575758

[5] Terzioğlu E, Arslan M, Balaban BG, Çakar ZP. Microbial silver resistance mechanisms: recent developments. World J Microbiol Biotechnol. 2022;38. 10.1007/s11274-022-03341-1 35821348

[6] Rewak-Soroczynska J, Dorotkiewicz-Jach A, Drulis-Kawa Z, Wiglusz RJ. Culture Media Composition Influences the Antibacterial Effect of Silver, Cupric, and zinc ions against Pseudomonas aeruginosa. Biomolecules. 2022;12. 10.3390/biom12070963 PMC9312735 35883519

[7] Tite T, Popa AC, Balescu LM, Bogdan IM, Pasuk I, Ferreira JMF et al. Cationic substitutions in hydroxyapatite: current status of the derived biofunctional effects and their in vitro interrogation methods. Materials. 2018;11. 10.3390/ma11112081 PMC6266948 30355975

[8] Mansoor S, Zahoor I, Baba TR, Padder SA, Bhat ZA, Koul AM et al. Fabrication of silver nanoparticles against fungal pathogens. Front Nanatechnol. 2021;3.

[9] Rozhin A, Batasheva S, Kruychkova M, Cherednichenko Y, Rozhina E, Fakhrullin R. Biogenic silver nanoparticles: synthesis and application as antibacterial and antifungal agents. Micromachines. 2021;12. 10.3390/mi12121480 PMC8708042 34945330

[10] Singh J, Vishwakarma K, Ramawat N, Rai P, Singh VK, Mishra RK et al. Nanomaterials and microbes’ interactions: a contemporary overview. 3 Biotech. 2019;9. 10.1007/s13205-019-1576-0 PMC6363610 30729092

[11] Rewak-soroczynska J, Sobierajska P, Targonska S, Piecuch A, Grosman L, Rachuna J (2021). New approach to antifungal activity of fluconazole incorporated into the porous 6‐anhydro‐α‐l‐galacto‐β‐d‐galactan structures modified with nanohydroxyapatite for chronic‐wound treatments—in vitro evaluation. Int J Mol Sci.

[12] Jiang Y, Yuan Z, Huang J (2020). Substituted hydroxyapatite: a recent development. Mater Technol.

[13] Lim PN, Shi Z, Neoh KG, Ho B, Tay B, Thian E (2014). The effects of silver, silicon-containing apatite towards bacteria and cell responses. Biomed Mater.

[14] da Silva JS, Machado TR, Trench AB, Silva AD, Teodoro V, Vieira PC et al. Enhanced photocatalytic and antifungal activity of hydroxyapatite/α-AgVO3 composites. Mater Chem Phys. 2020;252.

[15] Eraković S, Janković A, Ristoscu C, Duta L, Serban N, Visan A (2014). Antifungal activity of Ag:hydroxyapatite thin films synthesized by pulsed laser deposition on Ti and Ti modified by TiO 2 nanotubes substrates. Appl Surf Sci.

[16] Perlroth J, Choi B, Spellberg B (2007). Nosocomial fungal infections: epidemiology, diagnosis, and treatment. Med Mycol.

[17] Mendes CR, Dilarri G, Forsan CF, Sapata V, de MR, Lopes PRM, de Moraes PB et al. Antibacterial action and target mechanisms of zinc oxide nanoparticles against bacterial pathogens. Sci Rep. 2022;12. 10.1038/s41598-022-06657-y PMC8850488 35173244

[18] Sobierajska P, Nowak N, Rewak-Soroczynska J, Targonska S, Lewińska A, Grosman L (2021). Investigation of topography effect on antibacterial properties and biocompatibility of nanohydroxyapatites activated with zinc and copper ions: in vitro study of colloids, hydrogel scaffolds and pellets. Mater Sci Eng C.

[19] Salah I, Parkin IP, Allan E (2021). Copper as an antimicrobial agent: recent advances. RSC Adv.

[20] Sim W, Barnard RT, Blaskovich MAT, Ziora ZM. Antimicrobial silver in medicinal and consumer applications: a patent review of the past decade (2007–2017). Antibiotics. 2018;7. 10.3390/antibiotics7040093 PMC6315945 30373130

[21] Sudarsanan K, Young RA (1969). Significant precision in crystal structural details. Holly Springs hydroxyapatite. Acta Crystallogr Sect B: Struct Sci Cryst Eng Mater.

[22] Pazik R, Maczka M, Malecka M, Marciniak L, Ekner-Grzyb A, Mrowczynska L (2015). Functional up-converting SrTiO3:Er3+/Yb3 + nanoparticles: structural features, particle size, colour tuning and in vitro RBC cytotoxicity. Dalton Trans.

[23] Wiglusz RJ, Pazik R, Lukowiak A, Strek W (2011). Synthesis, structure, and Optical Properties of LiEu(PO3)4 nanoparticles. Inorg Chem.

[24] Anandalakshmi K, Venugobal J, Ramasamy V (2016). Characterization of silver nanoparticles by green synthesis method using Pedalium murex leaf extract and their antibacterial activity. Appl Nanosci (Switzerland).

[25] Targonska S, Rewak-Soroczynska J, Piecuch A, Paluch E, Szymanski D, Wiglusz RJ (2020). Preparation of a New Biocomposite designed for cartilage grafting with Antibiofilm Activity. ACS Omega.

[26] Wilcock CJ, Stafford GP, Miller CA, Ryabenkova Y, Fatima M, Gentile P (2017). Preparation and antibacterial properties of silver-doped nanoscale hydroxyapatite pastes for bone repair and augmentation. J Biomed Nanotechnol.

[27] Gottardo B, Lemes TH, Byzynski G, Paziani MH, von-Zeska-Kress MR, de Almeida MTG (2019). One-Pot synthesis and antifungal activity of nontoxic silver-loaded Hydroxyapatite Nanocomposites against Candida Species. ACS Appl Nano Mater.

[28] Ciobanu CS, Iconaru SL, Chifiriuc MC, Costescu A, Le Coustumer P, Predoi D. Synthesis and antimicrobial activity of silver-doped hydroxyapatite nanoparticles. Biomed Res Int. 2013;2013. 10.1155/2013/916218 PMC3591194 23509801

[29] Elbasuney S, El-Sayyad GS, Radwan SM, Correa-Duarte MA (2022). Antimicrobial, and Antibiofilm Activities of Silver Doped Hydroxyapatite: a Novel Bioceramic Material for Dental Filling. J Inorg Organomet Polym Mater.

[30] Rewak-Soroczynska J, Nowak N, Targonska S, Piecuch A, Wiglusz RJ (2022). The study of Nanosized Silicate-Substituted Hydroxyapatites co-doped with Sr2 + and Zn2 + ions related to their influence on Biological Activities. Curr Issues Mol Biol.

[31] Andes DR, Safdar N, Baddley JW, Alexander B, Brumble L, Freifeld A (2016). The epidemiology and outcomes of invasive Candida infections among organ transplant recipients in the United States: results of the Transplant-Associated infection Surveillance Network (TRANSNET). Transpl Infect Disease.

[32] Strollo S, Lionakis MS, Adjemian J, Steiner CA, Prevots DR (2017). Epidemiology of hospitalizations associated with invasive candidiasis, United States, 2002–2012. Emerg Infect Dis.

[33] Chen Y, Shi ZW, Strickland AB, Shi M. Cryptococcus neoformans infection in the Central Nervous System: the battle between host and Pathogen. J Fungi. 2022;8. 10.3390/jof8101069 PMC9605252 36294634

[34] Wirth F, Goldani LZ. Epidemiology of rhodotorula: An emerging pathogen. Interdisciplinary Perspectives on Infectious Diseases. 2012;2012. 10.1155/2012/465717 PMC3469092 23091485

[35] Thakur K, Singh G, Agarwal S, Rani L. MENINGITIS CAUSED BY RHODOTORULA RUBRA IN AN HUMAN IMMUNODEFICIENCY VIRUS INFECTED PATIENT. 2007. 10.4103/0255-0857.32730 17582194

[36] Jian Y, Chen X, Ahmed T, Shang Q, Zhang S, Ma Z (2022). Toxicity and action mechanisms of silver nanoparticles against the mycotoxin-producing fungus fusarium graminearum. J Adv Res.

[37] Targońska S, Wiglusz R. Studies of Luminescence Properties of Eu3 + Ions Doped the Silicate-Substituted Apatite and Co-doped with Strontium Ions. In: Light-Matter Interactions Towards the Nanoscale. NATO Science for Peace and Security Series B: Physics and Biophysics. 2022. p. 353–5.

[38] Targońska S, Szyszka K, Rewak-Soroczyńska J, Wiglusz R (2019). A new approach to spectroscopic and structural studies of the nano-sized silicate-substituted hydroxyapatite doped with Eu3 + ions. Dalton Trans.

